# Functional diversity of family 3 β-glucosidases from thermophilic cellulolytic fungus *Humicola insolens* Y1

**DOI:** 10.1038/srep27062

**Published:** 2016-06-08

**Authors:** Wei Xia, Yingguo Bai, Ying Cui, Xinxin Xu, Lichun Qian, Pengjun Shi, Wei Zhang, Huiying Luo, Xiuan Zhan, Bin Yao

**Affiliations:** 1 College of Animal Science, Zhejiang University, Hangzhou 310058, People’s Republic of China; 2 Key Laboratory for Feed Biotechnology of the Ministry of Agriculture, Feed Research Institute, Chinese Academy of Agricultural Sciences, Beijing 100081, People’s Republic of China; 3 Biotechnology Research Institute, Chinese Academy of Agricultural Sciences, Beijing 100081, People’s Republic of China

## Abstract

The fungus *Humicola insolens* is one of the most powerful decomposers of crystalline cellulose. However, studies on the β-glucosidases from this fungus remain insufficient, especially on glycosyl hydrolase family 3 enzymes. In the present study, we analyzed the functional diversity of three distant family 3 β-glucosidases from *Humicola insolens* strain Y1, which belonged to different evolutionary clades, by heterogeneous expression in *Pichia pastoris* strain GS115. The recombinant enzymes shared similar enzymatic properties including thermophilic and neutral optima (50–60 °C and pH 5.5–6.0) and high glucose tolerance, but differed in substrate specificities and kinetics. *Hi*Bgl3B was solely active towards aryl β-glucosides while *Hi*Bgl3A and *Hi*Bgl3C showed broad substrate specificities including both disaccharides and aryl β-glucosides. Of the three enzymes, *Hi*Bgl3C exhibited the highest specific activity (158.8 U/mg on *p*NPG and 56.4 U/mg on cellobiose) and catalytic efficiency and had the capacity to promote cellulose degradation. Substitutions of three key residues Ile48, Ile278 and Thr484 of *Hi*Bgl3B to the corresponding residues of *Hi*Bgl3A conferred the enzyme activity towards sophorose, and vice versa. This study reveals the functional diversity of GH3 β-glucosidases as well as the key residues in recognizing +1 subsite of different substrates.

The depletion of fossil fuel at enhanced rate and accompanied adverse effects on the global economic and environment has accelerated the research on its alternatives. Cellulosic materials like agricultural wastes and crop by-products (corn stover, wheat straw, bagasse, etc) are the most abundant polysaccharides in nature and represent the most valuable source of renewable energy^1^. Thus efficient utilization of cellulose biomass has been attracting attentions worldwide for the sustainable development and eco-efficiency^2^,^3^,^4^. Cost-effective process of enzymatic hydrolysis requires low production cost and highly active enzymes with great inhibitor tolerance and synergistic actions. In nature, complete hydrolysis of cellulose needs the synergistic action of a whole cellulolytic enzyme system, which includes endo-β-glucanase (EC 3.2.1.4), cellobiohydrolase (EC 3.2.1.91) and β-glucosidase (EC 3.2.1.21)^1^,^5^,^6^. β-Glucosidase can accelerate the decomposition of cellulose and improve the glucose yield by catalyzing the rate-limiting step of cellobiose hydrolysis^7^,^8^. The hyperproducing mutant strains of *Trichoderma reesei* are commercial producers of highly active cellulase (i.e. Celluclast 1.5 L, Novo Nodisk A/S, Bagsvaerd, Danmark), but have low β-glucosidase activities. Thus the Celluclast 1.5 L alone is inefficient in biomass degradation. To alleviate this limitation, it is a common practice to supplement other fungal β-glucosidase to avoid cellobiose inhibition and increase saccharification efficiency^8^. Thus, the discovery and biochemical characterization of novel fungal β-glucosidases are of great importance.

In general, β-glucosidases are hydrolases that acts upon β-bonds linking two glucose or glucose-substituted molecules (i.e., the disaccharide cellobiose or isoflavone aglycone). Based on amino acid sequences, the β-glucosidases are grouped into six families of glycoside hydrolase (GH), i.e. GH1, 3, 5, 9, 30 and 116 (http://www.cazy.org/)^9^,^10^. Besides biomass conversion, β-glucosidases are applied in other biological processes, such as biogenesis of various functional molecules (e.g., terpenols, flavonoids, phytohormones) from glycoside precursors^11^,^12^,^13^. Considering the great differences among homologous GH3 β-glucosidases^14^, an insight into the substrate specificity of β-glucosidases is beneficial for better utilization of this multifunctional biocatalyst. With the development of crystal determination, there have been several resolved GH3 β-glucosidase structures^15^,^16^,^17^. And several conserved substrate bind sites were verified in single protein by crystallisation of inhibitor complex or experimental determination, such as Arg156 and Tyr511 of GH3 β-glucosidase AaBGL1 from *Aspergillus aculeatus* (PDB: 4IIB)^15^, and Trp49 of β-glucosidase from *Aspergillus niger*^18^. But few researches were conducted to investigate the functional diversity and substrate specificity of multiple β-glucosidases from the same species.

Thermophilic filamentous fungi are excellent microbial sources of highly-active, thermostable β-glucosidases for industrial purposes^19^. To date, filamentous fungi including *Trichoderma*, *Aspergillus* and *Penicillium* are the main microbial sources of industrial cellulases^20^,^21^. Thermophilic *Humicola* spp. are also reported to have the ability to produce various cellulolytic enzymes^22^,^23^,^24^,^25^,^26^,^27^,^28^. However, the studies on β-glucosidases from this fungus appeared rather scanty. Up to now, an intracellular glucose- and xylose-stimulated β-glucosidase of GH1 (BglHi1) and a purified extracellular β-glucosidase of GH3 (BglHi2) from *H. insolens* have been biochemically characterized^24^,^25^. In this study, three GH3 β-glucosidase encoding genes (*Hibgl3A–C*) were cloned from *H. insolens* strain Y1 and successfully expressed in *Pichia pastoris* strain GS115. The enzymes were all most active under neutral and mesophilic conditions, but showed distinguished substrate specificity, catalytic efficiency and glucose tolerance. Further site-directed mutagenesis revealed the vital role of three residues in the substrate specificity of GH3 β-glucosidases.

## Results

### Gene cloning and sequence analysis

**Figure 1:**
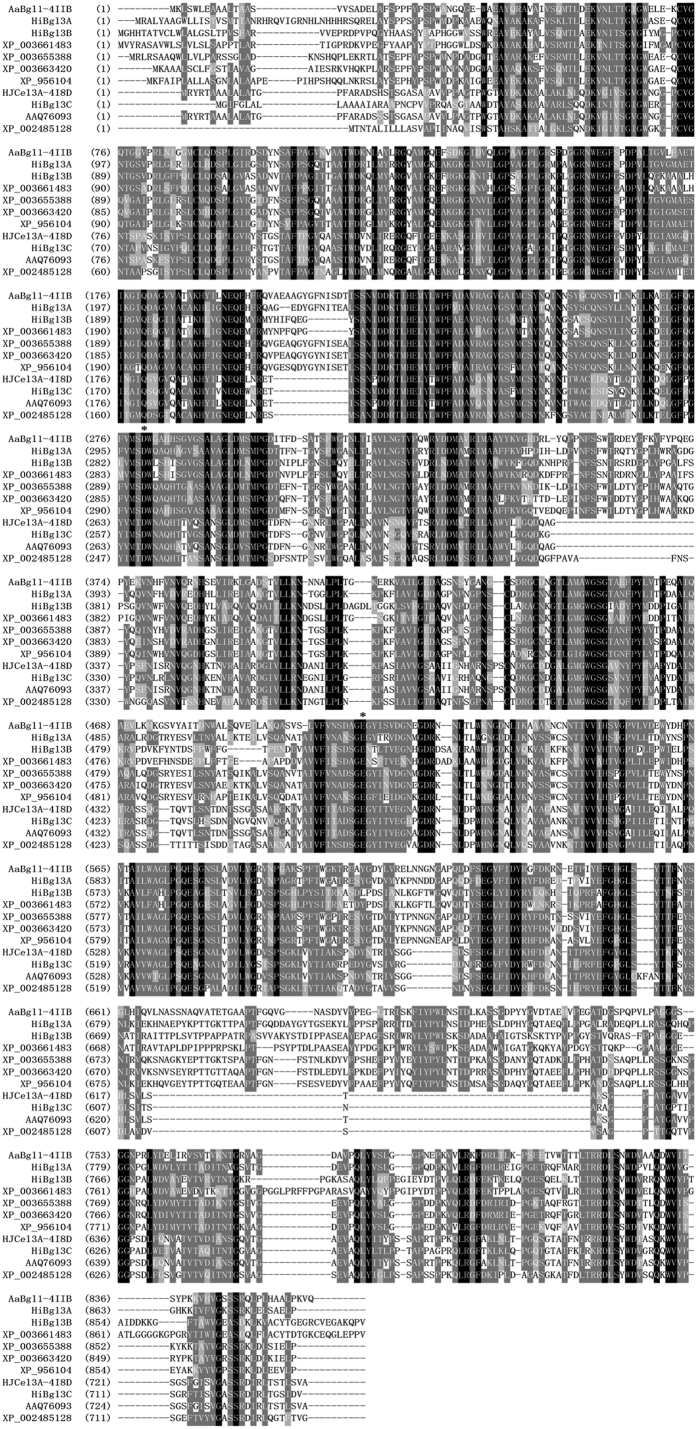
Sequence alignment of *Hi*Bgl3A, *Hi*Bgl3B and *Hi*Bgl3C with other GH3 β-glucosidases. The source and PDB codes or genebank accession numbers of these β-glucosidases are *Aspergillus aculeatus* (4IIB), *Hypocrea jecorina* (3ZYZ), *Myceliophthora thermophila* ATCC 42464 (XP_003663420), *Thielavia terrestris* NRRL 8126 (XP_003655388), *Neurospora crassa* OR74A (XP_956104), *Trichoderma viride* (AAQ76093) and *Talaromyces stipitatus* ATCC 10500 (XP_002485128). Identical and similar amino acids are indicated by black and gray shades, respectively. The putative catalytic residues were marked with asterisks. The three unique residues existed in HiBgl3B were framed by red rectangle.

Three β-glucosidase genes of GH3, i.e. *Hibgl3A*, *Hibgl3B*, and *Hibgl3C*, were cloned from the cDNA of *H. insolens* Y1. Their sequence information is summarized in Table 1. Although deduced *Hi*Bgl3A and *Hi*Bgl3B exhibit the highest identities with the GH3 protein from *Myceliophthora thermophila* ATCC 42464 (XP_003663420, 77% and 73%, respectively), they are only 39.9% identical to each other based on the analysis of AlignX from Vector NTI (Invitrogen). Deduced *Hi*Bgl3C is most similar to the GH3 β-glucosidase from *Trichoderma viride* (AAQ76093, 79% identity) and shared 35.4% and 37.3% sequence identity with *Hi*Bgl3A and *Hi*Bgl3B, respectively. Multiple sequence alignments of these three β-glucosidases with other GH3 proteins (see Fig. 1) indicated the conserved catalytic residues, Asp299 and Glu527 for *Hi*Bgl3A, Asp286 and Glu 515 for *Hi*Bgl3B, and Asp261 and Glu 463 for *Hi*Bgl3C, respectively.

### Expression and purification of recombinant β-glucosidases

The cDNA fragments of *Hibgl3A*, *Hibgl3B*, and *Hibgl3C* without the signal peptide-coding sequences were successfully expressed in *P. pastoris* GS115 with methanol induction in laboratory flasks. The β-glucosidase activities in the cultural supernatants of the recombinant strains harboring pIC9-*Hibgl3A*, pIC9-*Hibgl3B* and pIC9-*Hibgl3C* were 0.42, 0.76, and 3.54 U/ml, respectively. The crude enzymes were then concentrated, desalted and purified by anion exchange chromatography. SDS-PAGE analyses (see Supplementary Fig. S1) indicated the apparent molecular weights of purified *Hi*Bgl3A, *Hi*Bgl3B and *Hi*Bgl3C were about 120, 127 and 80 kDa, respectively. After deglycosylation with endo-β-*N*-acetylglucosaminidase H (Endo H), all enzymes showed reductions in the molecular masses, which were in agreement with their calculated values.

### Enzymatic properties

**Figure 2:**
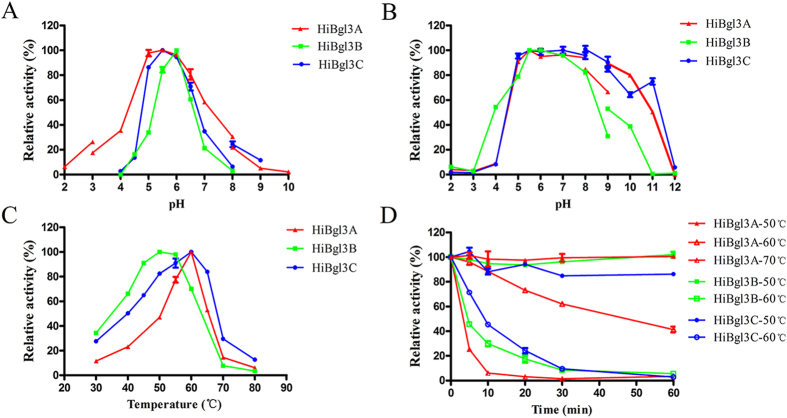
Enzymatic properties of the purified recombinant β-glucosidases using *p*NPG as the substrate. The relative activities of *Hi*Bgl3A (red triangle), *Hi*Bgl3B (green square) and *Hi*Bgl3C (blue circle) were plotted in the line chart. (**A**) Effect of pH on activities. (**B**) pH stability. (**C**) Effect of temperature on activities. (**D**) Thermostability at different temperatures. Each value in the panel represents the means ± SD (n = 3).

The enzymatic properties of *H. insolens* β-glucosidases were determined by using 4-nitrophenyl β-d-glucopyranoside (*p*NPG) as the substrate. All enzymes showed optimal activities at neutral pH, i.e. pH 5.5 for *Hi*Bgl3A and *Hi*Bgl3C and pH 6.0 for *Hi*Bgl3B (Fig. 2A). *Hi*Bgl3A and *Hi*Bgl3C retained stable (more than 80% activity) over the pH range of 5.0–10.0 at 37 °C for 1 h, while *Hi*Bgl3B was stable in a narrower range (pH 5.0–9.0) (Fig. 2B). When assayed under the pH optimum of each enzyme, *Hi*Bgl3A, *Hi*Bgl3B, and *Hi*Bgl3C exhibited maximum activities at 60, 50–55 and 60 °C, respectively (Fig. 2C). All enzymes were highly stable at 50 °C (Fig. 2D). The *H. insolens* β-glucosidases showed similar performance in the presence of 5 mM metal ions or chemical reagents tested, being activated by Mn^2+^, highly resistant to most metal ions, EDTA and β-mercaptoethanol, and sensitive to Ag^+^ and SDS (see Supplementary Table 1). Moreover, *Hi*Bgl3A and *Hi*Bgl3C retained more activities than *Hi*Bgl3B did in the presence of chemicals.

### Substrate specificity, kinetic parameters and inhibition constants

The substrate specificities of the three *H. insolens* β-glucosidases are shown in Table 2. When using disaccharides of different linkages as the substrate, the enzymes showed different preference, gentiobiose (β-1,6 linkage) > sophorose (β-1,2 linkage) > cellobiose (β-1,4 linkage) for *Hi*Bgl3A, sophorose > cellobiose > gentiobiose for *Hi*Bgl3C, respectively, and no *Hi*Bgl3B activity against all tested disaccharides. Aryl β-glycoside substrates (4-nitrophenyl compounds and soy isoflavones) that have a phenyl at subsite +1 were also tested. All enzymes showed much higher activities towards *p*NPG (over 8 fold) than against *p*NPC, *p*NPX, *p*NPGal and *p*NPAf, but varied in the hydrolysis of soy isoflavones. The activities of *Hi*Bgl3B and *Hi*Bgl3C against soy isoflavones followed the order of daidzin > genistin > glycitin, while *Hi*Bgl3A had relatively low but similar activities against the three tested soy isoflavone substrates. All enzymes had no observable activity on polysaccharides (barley β-glucan, sodium carboxymethylcellulose, Avicel, laminarin and lichenin).

The kinetics of *H. insolens* β-glucosidases on substrates *p*NPG and cellobiose are shown in Table 3. In comparison with the other two counterparts, *Hi*Bgl3C exhibited much higher substrate affinity (the lowest *K*_m_) and catalytic efficiency (*k*_*cat*_/*K*_*m*_). The glucose inhibition was also evaluated using *p*NPG as the substrate. *Hi*Bgl3B and *Hi*Bgl3C exhibited relatively high tolerance to glucose than that of *Hi*Bgl3A.

### Design, construction and specific activities determination of mutants

**Figure 3:**
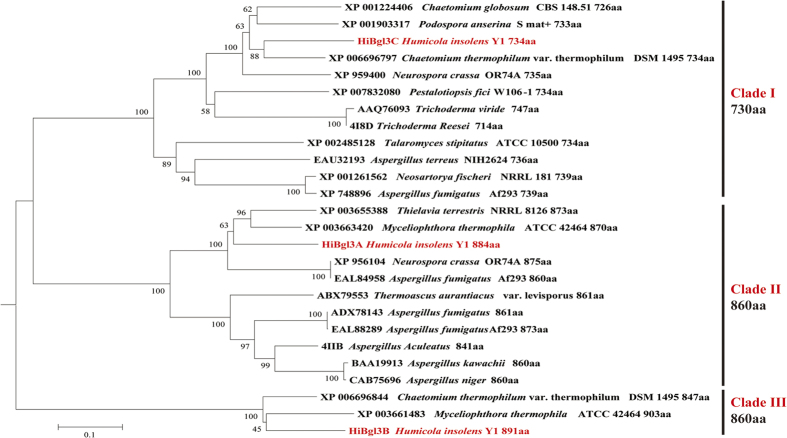
The phylogenetic tree generated from the analysis of *H. insolens* β-glucosidases and other closely related β-glucosidases amino acid sequences in the NCBI database using the Neighbor-Joining method. The numbers on nodes correspond to the percentage bootstrap values for 1,000 replicates. The accession number of each SOD in GenBank is labelled prior to the species name.

Three isoenzymes exhibited distinct features in terms of substrate specificity. To investigate the evolutionary relationship of GH3 β-glucosidases, a phylogenetic analysis on the amino acid sequences of *H. insolens* β-glucosidases and counterparts obtained from the NCBI database using the Neighbor-Joining (NJ) method (shown in Fig. 3) indicated that *Hi*Bgl3A, *Hi*Bgl3B and *Hi*Bgl3C belonged to different evolutionarily related clades. Those belonging to clade II and III have similar length, about 860 amino acids, with different substrate specificities. Sequence alignment suggested that three distinct unique residues (framed by red rectangle in Fig. 1) existed in *Hi*Bgl3B and the homologous protein XP_003661483, i.e. Ile48, Ile278 and Thr484, which were generally Trp, Phe, and Tyr, respectively, in most GH3 β-glucosidases. These three residues are all located at the entrance of the enzyme’s catalytic pocket, and may relate to substrate specificity. Thus we conducted site-directed mutagenesis on *Hi*Bgl3A and *Hi*Bgl3B of similar lengths to verify the impact of these three sites on recognizing +1 subsite of different substrates. The specific activities and kinetic values of mutants towards aryl β-glycoside *p*NPG and three disaccharides were measured (shown in Table 4). Although the single and combined mutants I48W, I278F, T484Y and I48W/I278F/T484Y of *Hi*Bgl3B showed decreased activities towards *p*NPG, all the mutants conferred obvious hydrolysis activities on sophorose, which were much higher than the activities on *p*NPG of their own. However, no activity on the other two disaccharides was detected. In contrast, negative mutants of *Hi*Bgl3A all lost hydrolysis activities towards disaccharides, and the mutants W69I and F304I even became completely inactivated towards *p*NPG (data not shown). The turnover numbers (*k*_cat_) of *Hi*Bgl3B mutants all decreased substantially while no significant change was found in the *K*_m_ values, leading to declines in catalytic efficiency. However, introduction of mutation Y509T caused the increase of the *K*_m_ value, but did not affect the *k*_cat_ value.

#### Enzymatic saccharification of cellulose materials

**Figure 4:**
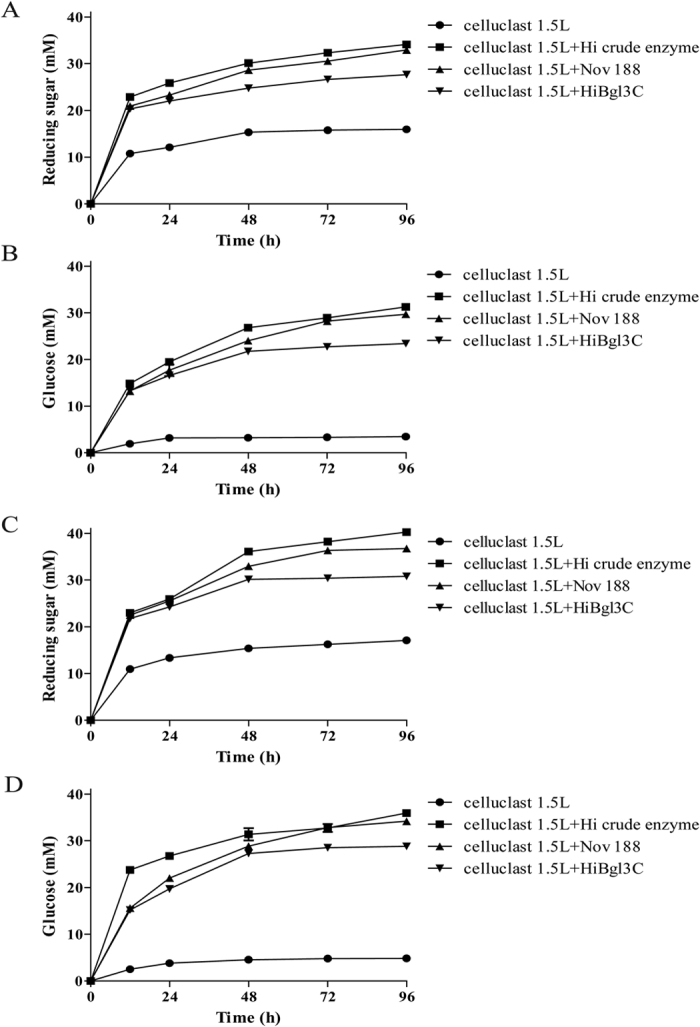
Enzymatic saccharifications of pretreated cellulose materials. The concentrations (mM) of reducing sugar and glucose in the hydrolyzates of corn stover and Avicel by different enzyme combinations (cellulase at 5 FPU/g and β-glucosidase at 11 BGU/g, resepctivley) were shown in (**A**–**D**), respectively. Circle: Celluclast 1.5 L only; square: Celluclast 1.5 L plus crude enzyme of *H. insolens* Y1; upper triangle: Celluclast 1.5 L plus Novozyme 188; lower triangle: Celluclast 1.5 L plus *Hi*Bgl3C.

To investigate the application potential of the most efficient *H. insolens* β-glucosidase *Hi*Bgl3C in biomass conversion, the sacchrification efficiencies of different enzyme combinations using corn stover and Avicel as the cellulosic materials were compared as shown in Fig. 4. Over 96 h incubation at pH 5.5 and 50 °C, supplementation of the commercial *T. reesei* cellulase Celluclast 1.5 L at the dosage of 5 FPU per gram dry materials (DM) released 15.95 mM and 17.09 mM of reducing sugars from corn stover and Avicel, respectively, in which glucose accounted for 3.46 (21%) and 4.85 mM (28%), respectively. The culture supernatants of *H. insolens* Y1 showed considerable ability to decompose cellulose materials with the activities of 4 FPU/ml and 30 BGU/ml. When supplemented the crude supernatants of *H. insolens* Y1 (at the dosage of 11 BGU/g DM of β-glucosidase) into commercial Celluclast 1.5 L, the amounts of reducing sugar and yields of fermentable sugars were increased substantially. Their synergistic action resulted in the increased release of reducing sugars from corn stover and Avicel (31.43 and 40.28 mM, respectively) and increased glucose conversion rate (percentage of glucose amount to that of reducing sugars, 99% for corn stover and 77% for Avicel, respectively). When substituted the culture supernatants of *H. insolens* Y1 with commercial Novozyme 188 (Sigma-Aldrich) at the same dosage of β-glucosidase (11 BGU/g DM), two-fold increase of saccharification efficiency was achieved. As results, 32.91 mM and 36.76 mM of reducing sugars were released from corn stover and Avicel, respectively, and the glucose conversion rates were higher than 90%. *Hi*Bgl3C showed comparable performance to Novozyme 188 in saccharification, releasing more than 80% of the reducing sugars of Novozyme 188 (27.67 mM and 28.83 mM, respectively) and converting 84% and 93% of the reducing sugars into glucose for corn stover and Avicel, respectively.

## Discussion

The fungus *H. insolens* is one of the most powerful decomposers of crystalline cellulose and represents a potential industrial producer of cellulases^19^,^23^,^27^. However, the studies on *H. insolens* β-glucosidases are much limited to only two reports^24^,^25^, and the inner peptide sequences of the purified GH3 β-glucosidase BglHi2^25^ showed 100% sequence identity to *Hi*Bgl3A of the present work. Although heterologous expression of cellulolytic enzymes is becoming the research focus^29^, none of the β-glucosidase genes has been cloned from *H. insolens* Y1 or heterologously expressed as well as functionally characterized. In this study, three GH3 β-glucosidase genes were identified based on the partial genome sequence of *H. insolens* Y1, and their functions and saccharification performance were revealed after heterologous expressions in *P. pastoris* GS115. Although the three protein sequences were discrepant and grouped into three different evolutionary clades, their biochemical properties had some similarities. For example, *Hi*Bgl3A, *Hi*Bgl3B and *Hi*Bgl3C exhibited maximum activities at 50–60 °C and pH 5.0–6.0. In contrast, most β-glucosidases from filamentous fungi, i.e. *Trichoderma* and *Aspergillus*, have acidic pH optimal in the range of pH 4.0–5.0 (Table 2)^30^,^31^,^32^,^33^,^34^,^35^,^36^.

The three β-glucosidases from *H. insolens* Y1 exhibited distinct difference in the substrate specificity towards disaccharides and aryl β-glycosides. *Hi*Bgl3A and *Hi*Bgl3C are typical GH3 β-glucosidases with strong affinities to a wide range of substrates including both disaccharides and aryl-glycosides although the performances are not the same. It might due to the distinct structures of these two β-glucosidases with inhomogeneous protein sequences, which were grouped into different evolutionary clades. The activities of these two enzymes on cellobiose are higher than that of many fungal counterparts^41^,^42^,^43^ including that from *T. reesei* (*Hypocrea jecorina*, 21 U/mg)^33^. However, *Hi*Bgl3B has no activity on all tested disaccharides and only showed considerable hydrolysis ability on *p*NPG. It’s similar to the BglF from *Aspergillus oryzae* that has no activity on oligosaccharides (<0.01 U/mg) and a very low specific activity on *p*NPG (0.6 U/mg)^14^. This kind of β-glucosidase is defined as aryl β-glucosidase, and widely exists in microorganisms^44^. A previous study has reported that GH3 β-glucosidases have a strict stereochemical requirement to accommodate β-D-glucopyranose at subsite −1, while subsites + seem insignificant in both substrate binding and hydrolysis^10^. This relative plasticity at subsite +1 might account for the broad substrate specificity of GH3 β-glucosidases towards different aglycon structures. Besides, β-glucosidases of different sequence clades may vary in conformation at the subsite +1, consequently leading to variations in the activities on substrates with different aglycons at this site. Based on the multiple sequence alignment and structure analysis, three conserved substrate recognizing residues for the subsite +1 of cellobiose were identified in the β-glucosidases capable of hydrolyzing cellobiose^15^,^17^, for instance, Trp68, Phe305 and Tyr511 of *Aa*BGL1 from *Aspergillus aculeatus* (PDB: 4IIB), were substituted by Ile48, Ile278 and Thr484 of *Hi*Bgl3B, respectively (see Fig. 1)^15^. The lack of ability to interact with the second sugar ring of disaccharides might be responsible for its unique substrate specificity. It could be speculated that this sort of β-glucosidases does not contribute to the deconstruction of cellulose, though the natural function is unclear yet. This difference indicates their functional diversity.

**Figure 5:**
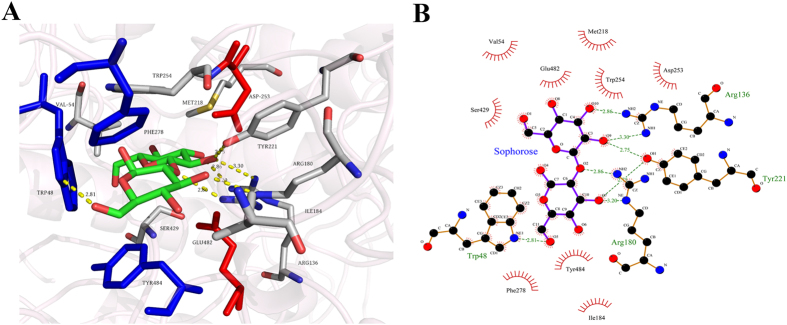
The stereoview of the catalytic pocket of docked model of mutant I48W/I278F/T484Y combining with sophorose (**A**) and schematic plan of interaction analyses between the ligand and residues (**B**). Sophorose (green) and several key residues (red for catalyst and blue for W48, F278 and Y484) are depicted as sticks. Ligand–protein interactions were obtained using Ligplot^+^.

Activity changes between wild type and mutant enzymes suggested that Ile48, Ile278 and Thr484 have effects on the substrate recognition of enzymes. To investigate the interactions between I48, I278 and T484 and sophorose, a molecular docking was performed, and the results were shown in Fig. 5A. The interactions were analyzed by software Ligplot^+^ and shown in the planar graph Fig. 5B. W48, F278 and Y484 are supposed to interact with ligand sophorose at the subsite +1 by hydrophobic and polar interactions, which are absent in wild type *Hi*Bgl3B. And compared to other two disaccharides β-1-4-cellobiose and β-1-6-gentiobiose, the hydroxyl at the C6 site of +1 subunit of β-1-2-sophorose was outward, thus requiring smaller spatial position to locate into the binding pocket. It may explain why *Hi*Bgl3B mutants had no activity on cellobiose and gentiobiose. In contrast, wild type *Hi*Bgl3B is able to combine the highly hydrophobic aromatic ring at its +1 binding site. However, the binding affinity seems to vary significantly among different sequences and structures because the same case did not apply to the negative mutants of HiBgl3A. For example, the single mutants of *Hi*Bgl3A had no activity towards disaccharides, and no accumulated effect was found in the combination mutant of *Hi*Bgl3B. We conjecture that these three residues in *Hi*Bgl3A must have an integral role in the binding with disaccharide substrates. Without Trp69 and Phe304, the enzyme lost hydrolysis activities on both aryl aglycones and disaccharides, indicating the indispensable role played by these two residues. On the other hand, *Hi*Bgl3A and *Hi*Bgl3B share only 39.9% sequence identity, which may accounts for the huge differences in protein structure, especially the dramatic difference at substrates recognition sites. The disordered conformations of *Hi*Bgl3B mutants or abnormal binding to *p*NPG is also supported by the sharp decline of *k*_cat_ values. The changes in the *K*_m_ values of wild type enzymes and mutants also provided proofs of quite distinct effect of these three sites in different clade of β-glucosidases. As for the current job, they all contributed to the binding of disaccharide substrates for *Hi*Bgl3A and *Hi*Bgl3B. The only difference is that they had no effect on the recognition of *p*NPG for *Hi*Bgl3B while affected the recognition of *Hi*Bgl3A.

Moreover, *H. insolens* β-glucosidases also exhibited diversity in kinetics and glucose tolerance. Most of the microbial β-glucosidases that have the ability to hydrolyze cellobiose are very sensitive to glucose (with the *K*_*i*_ value in the range of 0.35–10 mM glucose, shown in Table 2). The three *H. insolens* β-glucosidases had different inhibition constants of glucose: *Hi*Bgl3B (55.2 mM) was most resistant to glucose, followed by *Hi*Bgl3C (37.1 mM) and *Hi*Bgl3A (25.0 mM) using *p*NPG as the substrate. Their *K*_*i*_ values were much higher than most fungal GH3 β-glucosidases^30^,^31^,^32^,^33^,^34^,^35^, including the most generally used commercial β-glucosidase Novozyme 188(*K*_*i*_ value of 2.7 mM)^30^. In addition, *Hi*Bgl3A and *Hi*Bgl3C had higher *k*_*cat*_/*K*_*m*_ values (11.1/s/mM and 23.0/s/mM, respectively) towards cellobiose than that of several other β-glucosidases^35^,^38^,^40^, but lower than that of Novozyme 188 (36/s/mM)^30^. A saccharification experiment was carried out to evaluate the ability of the most efficient *Hi*Bgl3C in promoting cellulose degradation. Given the same conditions, supplementation of *Hi*Bgl3C yielded more than 80% of the reducing sugars released by commercial Celluclast 1.5 L and Novozyme 188, and achieved almost equivalent glucose conversion rate. It was reported that most microbial β-glucosidases performed worse in synergetic cellulose degradation than Novozyme 188 because of their low inhibition constants^7^. *Ta*BG3 from *Acremonium thermophilum* and *At*BG3 from *Thermoascus aurantiacus* had good performances by exhibiting *k*_cat_/*K*_m_ values of 26.2 kJ · mol^−1^ and 20.5 kJ · mol^−1^ at 55 °C, respectively, which were also lower than the value of Novozyme 188 (29.5 kJ · mol^−1^)^7^. However, NpaBGS from the buffalo rumen fungus Neocallimastix patriciarum W5 had a slightly better efficiency than Novozyme 188 at a lower temperature 40 °C^39^. It demonstrated that, with outstanding cooperation effect, *Hi*Bgl3C from *H. insolens* Y1 has great application potential to enhance the enzymatic saccharification of biomass.

## Conclusions

Three *H. insolens* β-glucosidases of GH3 were produced in yeasts and systematically characterized. The enzymes are highly tolerant to glucose inhibition (25.0–55.2 mM) and share thermophilic and neutral features, but vary in substrate specificities. *Hi*Bgl3A and *Hi*Bgl3C are active towards both cellobiose and aryl β-glucoside while *Hi*Bgl3B is a typical aryl β-glucosidase. Substitutive mutations proved the vital role of three key residues in recognizing +1 subsite of different substrates. Their catalytic performances showed difference in biological processes. This work revealed the relationship between sequence differences and functional diversity.

## Methods

### Strains, media, vectors and chemicals

*H. insolens* Y1 GCMCC 4573 was routinely cultured in the wheat bran medium^45^. *Escherichia coli* Trans1-T1 and vector pEASY-T3 (TransGen, Beijing, China) were used for gene cloning. The gene expression vector and heterologous expression host were pPIC9 and *P. pastoris* GS115 (Invitrogen, Carlsbad, CA), respectively. The DNA purification kit, restriction endonucleases and *LA Taq* DNA polymerase were purchased from TaKaRa (Otsu, Japan). T4 DNA ligase and the total RNA isolation system kit were purchased from Promega (Madison, WI). The cDNA synthesis kit was purchased from TransGen. Barley β-glucan, Avicel, 4-nitrophenyl β-d-glucopyranoside (*p*NPG), 4-nitrophenyl β-d-xylopyranoside (*p*NPX), 4-nitrophenyl α-l-arabinofuranoside (*p*NPAf), 4-nitrophenyl α-d-galactopyranoside (*p*NPGal), 4-nitrophenyl α-l-arabinopyranoside (*p*NPAb), 4-nitrophenyl β-d-cellobioside (*p*NPC), disaccharides cellobiose, sophorose and gentibiose and soybean flavones daidzin, genistin and glycitin were all purchased from Sigma-Aldrich. Sodium carboxymethylcellulose (CMC-Na), laminarin and lichenin were obtained from Megazyme (Wicklow, Ireland). All other chemicals used were of analytical grade and commercially available.

#### Gene cloning and sequence analysis

The total RNA of *H. insolens* Y1 was extracted from the mycelia after3 days’ growth in the inducing wheat bran medium, and was reverse transcribed into cDNA by TransScript^®^ One-Step gDNA Removal and cDNA Synthesis SuperMix kit (TransGen). The gene fragments without the signal peptide-coding sequence were amplified using *H. insolens* Y1 cDNA as the template, at an annealing temperature of 60 °C, with three specific primer sets (shown in Supplementary Table S2). The PCR products were purified and ligated into the pEASY-T3 vector for sequencing. Vector NTI Advance 10.0 software (Invitrogen) was used to analyze the DNA sequence and to predict the molecular weight and *p*I of proteins and perform multiple sequence alignments. The signal peptide and the potential *N*-glycosylation sites were predicted by the SignalP 4.1 server (http://www.cbs.dtu.dk/services/SignalP/) and the NetNGlyc 1.0 Server (http://www.cbs.dtu.dk/services/NetNGlyc/), respectively. The neighbor-joining phylogenetic tree based on the coding sequences of was performed by using MEGA (Version 6.0).

#### Expression and purification of the recombinant enzymes

The cDNA fragments without the signal peptide-coding sequences and the pPIC9 vector were both digested by *Spe*I and *Not*I (for *Hibgl3A*) or *EcoR*I and *Not*I (for *Hibgl3B* and *Hibgl3C*) and ligated into in-frame fusion of the α-factor signal peptide to construct the recombinant plasmids. The recombinant plasmids were linearized using *Bgl*II and transformed into *P. pastoris* GS115 competent cells by electroporation using Gene Pulser X cell Electroporation System (Bio-Rad). Minimal dextrose medium (MD) plates were prepared for the screening of positive transformants. The positive transformants were transferred to buffered glycerol complex medium (BMGY) and grown at 30 °C for 2 days. The cells were collected by centrifugation and resuspended in buffered methanol complex medium (BMMY) containing 0.5% methanol for induction. The β-glucosidase activities of the culture supernatants were assayed using *p*NPG as the substrate, and the transformants exhibiting the highest β-glucosidase activities were subjected to high level expression in 1-l Erlenmeyer flasks. The culture supernatants of aforementioned recombinant strain were collected by centrifugation (12,000× *g*, 4 °C, 10 min), followed by concentration through a Vivaflow ultrafiltration membrane (Vivascience) with a molecular weight cut-off of 5 kDa. The crude enzyme was loaded onto a FPLC HiTrap Q Sepharose XL 5 mL column (GE Healthcare) that was equilibrated with 20 mM Tris-HCl (pH 8.0). Proteins were eluted using a linear gradient of NaCl (0–1.0 M) in the buffer mentioned above at a flow rate of 3.0 ml/min. Fractions exhibiting β-glucosidase activities were pooled and subjected to SDS-PAGE analysis. The protein concentration was determined by a Bradford assay with bovine serine albumin as a standard. To remove *N*-glycosylation during heterologous expression in *P. pastoris*, proteins were incubated with 500 U of Endo H at 37 °C for 2 h according to the manufacturer’s instructions (New England Biolabs). The deglycosylated enzymes were also analyzed by SDS-PAGE.

#### Enzyme activity assay

The β-glucosidase activities were assayed using *p*NPG and cellobiose as the substrate. One unit of β-glucosidase activity was defined as the amount of enzyme that released 1 μmol of products per minute under the assay conditions. For substrate *p*NPG, the standard reaction system consisted of 250 μl of appropriately diluted enzyme and 250 μl of McIlvaine buffer containing 2 mM *p*NPG. After incubation at a certain temperature for 10 min, 1.5 mL of 1.0 M Na_2_CO_3_ was added into the system to terminate the reaction. The amount of *p*-nitrophenol released was determined spectrophotometrically by reading the absorbance at 405 nm. Each experiment was performed in triplicate. And for cellobiose, the standard reaction was carried out with 70 μl of appropriately diluted enzyme and 70 μl of McIlvaine buffer containing 2 mM cellobiose for 10 min followed by a boiling water bath to terminate the reaction. GOD-POD coloring solution (2.1 ml) was then added into the system, and the absorbance at 520 nm was determined to calculate the amount of released glucose.

#### Biochemical characterization

The optimal pH for β-glucosidases activities were determined at respective appropriate temperature for 10 min over a pH range of 2.0–11.0. Buffers used for the assays were as following: 100 mM Na_2_HPO_4_-citric acid (pH 2.0–8.0), 100 mM Tris-HCl (pH 8.0–9.0), and 100 mM glycine-NaOH (pH 9.0–11.0). To estimate pH stability, the enzymes were pre-incubated in the buffers mentioned above without substrate at 37 °C (physiological temperature) for 1 h, and the residual activities were measured under standard conditions (pH 6.0, 60 °C, and 10 min). The optimal temperatures were examined at the optimal pH by measuring the enzyme activity over the temperature range of 30 and 90 °C. The thermostability was investigated by measuring the residual enzyme activities after preincubation of the enzymes at 60 °C or 70 °C and optimal pH without substrate for various periods. The samples were collected at 0, 2, 5, 10, 20, 30 and 60 min, respectively. Enzyme activity assays were performed under the standard conditions. The activities of β-glucosidases were measured in the presence of 5 mM of various metal ions and chemical reagents (Ag^+^, Ca^2+^, Li^+^, Co^2+^, Cr^3+^, Ni^2+^, Cu^2+^, Mg^2+^, Fe^3+^, Mn^2+^, Hg^2+^, Pb^2+^, EDTA, SDS or β-mercaptoethanol) to estimate their effects on enzymes. The reaction without any additive was used as a blank control.

#### Substrate specificity

To investigate the substrate specificity of *Hi*Bgl3A, *Hi*Bgl3B and *Hi*Bgl3C, polysaccharides (barley β-glucan, sodium carboxymethylcellulose, Avicel, laminarin, and lichenin), disaccharides (cellobiose, sophorose, and gentiobiose), artificial *p*-nitrophenyl derivatives (*p*NPG, *p*NPAf, *p*NPX, *p*NPGal, *p*NPAb, *p*NPC) and soybean flavones (daidzin, genistin, and glycitin) were each used as the substrate to determine the corresponding activities at a final concentration of 1% (w/v) or 1 mM.

#### Kinetic parameters and glucose inhibition

The *K*_*m*_, *V*_*max*_ and *k*_*cat*_ values of *H. insolens* β-glucosidases were determined under each optimal conditions for 5 min in 100 mM Na_2_HPO_4_-citric acid containing 1–10 mM *p*NPG or cellobiose as the substrate. The data were plotted according to the Lineweaver-Burk method. To estimate the glucose inhibition, the β-glucosidase activities were determined at two final concentrations of *p*NPG (0.75 mM and 1 mM) in the presence of different amounts of glucose (5–50 mM) using the standard activity assay. The data were analyzed by a Dixon plot to calculate the inhibition constant (*K*_*i*_) value of glucose.

#### Homology-modeling and molecular docking of H. insolens β-glucosidases

The tertiary structure of deduced β-glucosidases were homology-modeled using the Discovery Studio v2.5 software (Accelrys) with the crystal structure of AaBGL1 from *A. aculeatus* (PDB: 4IIB) as the template for *Hi*Bgl3A and *Hi*Bgl3B, and Cel3A from *Hypocrea jecorina* for *Hi*Bgl3C. The modeled structures were then refined by embedded program energy minimization and Verify-3D. The molecular docking of modeled mutant *Hi*Bgl3B-I48W/I278F/T484Y and β-1,2-sophorose was carried out by AutoDock Vina^10^ to elucidate the substrate binding interaction. The docking grid with an appropriate size of 40 × 40 × 40 of 0.375 Å spacing was set to center the β-carbon atom of Asp252 of *Hi*Bgl3B. Three-dimensional molecular visualization and figure preparation were performed with PyMOL version 1.7.2.1 (The PyMOL Molecular Graphics System).

#### Construction and expression of mutants

All mutants were constructed using overlap extension PCR based on the structural analyses of *Hi*Bgl3A and *Hi*Bgl3B. Several primer sets (shown in Supplementary Table S2) for site-directed mutagenesis were synthesized. Expression and purification of the mutants were conducted as the same with wild type enzymes.

### Enzymatic saccharification

Two cellulose materials, corn stover and Avicel, were pretreated by 1% NaOH solution at 121 °C for 30 min in an autoclave sterilizer, washed with ddH_2_O, dried and mixed with 20 ml of 100 mM Na_2_HPO_4_-citric acid buffer (pH 5.5) in 50 ml shake flasks. Three enzyme combinations, i.e. Celluclast 1.5 L supplemented with the crude enzyme of *H. insolens* Y1, Novozyme 188, and *Hi*Bgl3C, respectively, with the total activities of 5 FPU and 11 BGU per gram dry materials (DM) were added into the cellulose mixture and incubated at 50 °C and 200 rpm in a shaking bath for 96 h. Celluclast 1.5 L alone was used as the control group. Hydrolyzates were collected at several intervals and centrifuged at 12,000 rpm, 4 °C for 10 min. The amounts of reducing sugars and glucose released in the supernatants were determined using the DNS^46^ and GOD-POD methods, respectively. All these experiments were conducted with three replicates.

## Additional Information

**Accession codes:** The cDNA sequences have been submitted to GenBank, and accession numbers are KT203370, KT203372 and KT203372 for Hibgl3A, Hibgl3B, and Hibgl3C, respectively.

**How to cite this article**: Xia, W. *et al.* Functional diversity of family 3 β-glucosidases from thermophilic cellulolytic fungus *Humicola insolens* Y1. *Sci. Rep.* **6**, 27062; doi: 10.1038/srep27062 (2016).

## Supplementary Material

Supplementary Information

## Figures and Tables

**Table 1:** Sequence information of the three GH3 β-glucosidases from *H. insolens* Y1.

	HiBgl3A	HiBgl3B	HiBgl3C
cDNA (bp)	2655	2676	2205
Deduced amino acids (aa)	884	891	734
Putative signal peptide (aa)^a^	20	23	17
Theoretical molecular mass (kDa)^b^	95.1	94.6	78.4
Estimated *p*I^b^	5.93	6.03	6.26
Putative *N*-glycosylation sites^b^	12	7	2

^a^Predicted by the SignalP 4.1 server (http://www.cbs.dtu.dk/services/SignalP/).

^b^Estimated by the Vector NTI Advance 10.0 software (Invitrogen).

^c^Predicted by the NetNGlyc 1.0 Server (http://www.cbs.dtu.dk/services/NetNGlyc/).

**Table 2:** Substrate specificity of the three *H. insolens* β-glucosidases.

Substrate^a^	Specific activity (U/mg)		
	HiBgl3A	HiBgl3B	HiBgl3C
Disaccharide			
Cellobiose	36.3 ± 0.2	ND^b^	56.4 ± 1.2
Sophorose	94.6 ± 0.9	ND	113.9 ± 0.6
Gentiobiose	103.4 ± 1.6	ND	50.4 ± 0.5
Aryl β-glycoside			
*p*NPG	57.5 ± 0.3	31.6 ± 0.6	158.8 ± 2.0
*p*NPC	3.5 ± 0.2	1.7 ± 0.2	17.8 ± 0.2
*p*NPX	1.6 ± 0.1	0.8 ± 0.2	4.1 ± 0.2
*p*NPGal	1.3 ± 0.1	0.8 ± 0.3	1.9 ± 0.2
*p*NPAf	3.1 ± 0.2	ND	ND
Daidzin	20.4 ± 0.5	62.6 ± 2.3	80.0 ± 1.7
Genistin	25.5 ± 0.8	28.6 ± 0.4	22.1 ± 0.1
Glycitin	19.0 ± 1.1	15.9 ± 0.8	8.8 ± 0.5

^a^The final concentration of each substrate is 1 mM.

^b^ND, not detected.

**Table 3:** Property comparison of microbial β-glucosidases^a^.

Species	Enzymes	Optimum		*K*_*m*_ (mM)		*k*_*cat*_/*K*_*m*_ (/s/mM)		*K*_*i*_ (mM)		References
		pH	T (°C)	*p*NPG	CB	*p*NPG	CB	*p*NPG	CB	
*Humicola insolens* Y1	*Hi*Bgl3A	5.5	60	0.90	8.44	81.6	11.1	25.0	–	This work
*H. insolens* Y1	*Hi*Bgl3B	6.0	50	1.51	–	28.6	–	55.2	–	This work
*H. insolens* Y1	*Hi*Bgl3C	5.5	55	0.20	6.63	1557	23.0	37.1	–	This work
*Aspergillus niger*	N188	4.8	50	0.57	0.88	41	36	2.7	–	30
*Aspergillus foetidus*		4.8	65	0.41	–	–	–	8.1	–	31
*Aspergillus japonicus*		5.0	40	0.60	0.95	432	368	2.73	–	32
*Aspergillus oryzae*		5.0	50	0.29	1.96	1270	510	2.9	5	33
*Fomitopsis palustris*		5.0	50	0.12	4.8	6160	21	0.35	–	34
*Hypocrea jecorina*		5.0	50	0.09	0.75	466	157	0.51	–	32
*Myceliophthora thermophila*		5.0	40	0.39	2.64	376	17	0.28	–	35
*Trichoderma reesei*		4.5	50	–	0.54	–	41	–	0.29	36
*Thermoanaerobacterium thermosaccharolyticum* DSM 571	rBGL	6.4	70	0.63	7.9	–	13.3	600	–	37
Uncultured bacterium		6.0	40	0.39	20.4	–	0.65	1000	–	38
*Neocallimastix patriciarum*	*Npa*BGS	6.0	40	–	–	–	–	–	–	39
Cow rumen metagenome	LAB25g2	5.2	50	0.45	4.88	0.92	0.2	–	–	40

^a^*p*NPG was used for the determination of optimal conditions, and *p*NPG and cellobiose (CB) were used for the determination of kinetics and glucose inhibition.

**Table 4:** Specific activities and kinetic values of the wild type and mutants of HiBgl3A and HiBgl3B.

Enzymes	Specific activity (U/mg)				Kinetic parameter on *p*NPG			
	*p*NPG	Cellobiose	Gentiobiose	Sophorose	*K*_m_ (mM)	*V*_max_ (μmol/min/mg)	*k*_cat_ (/s)	*k*_cat_/*K*_m_ (/s/mM)
HiBgl3A								
Wild type	57.5 ± 0.3	36.3 ± 0.2	103.4 ± 1.6	94.6 ± 0.9	0.90	46.2	73.08	81.56
Y509T	22.3 ± 2.4	ND^a^	ND	ND	2.45	43.0	68.16	27.82
HiBgl3B								
Wild type	31.6 ± 0.6	ND	ND	ND	1.51	25.5	40.42	26.77
I48W	0.95 ± 0.8	ND	ND	3.70 ± 1.7	1.99	2.03	3.21	1.61
I278F	2.68 ± 1.4	ND	ND	3.59 ± 0.4	2.42	6.07	9.61	3.98
T484Y	4.46 ± 0.9	ND	ND	5.64 ± 0.7	2.37	9.51	15.05	6.36
I48W/I278F/T484Y	1.0 ± 0.7	ND	ND	3.36 ± 0.5	2.48	2.68	4.24	1.71

^a^ND, not detected.
