# Supplementary material for: Functional diversity of family 3 β-glucosidases from thermophilic cellulolytic fungus Humicola insolens Y1
Source: Sci Rep. 2016 Jun 8;6:27062. doi: 10.1038/srep27062 (PMC4897640; doi:10.1038/srep27062)

Functional diversity of family 3 β-glucosidases from thermophilic cellulolytic fungus *Humicola insolens* Y1

Wei Xia[1,2*], Yingguo Bai[2*], Ying Cui[2], Xinxin Xu[3], Lichun Qian[1], Pengjun Shi[2], Wei Zhang[3], Huiying Luo[2], Xiuan Zhan[1] & Bin Yao[2]

[1] College of Animal Science, Zhejiang University, Hangzhou 310058, People's Republic of China. [2] Key Laboratory for Feed Biotechnology of the Ministry of Agriculture, Feed Research Institute, Chinese Academy of Agricultural Sciences, Beijing 100081, People's Republic of China. [3] Biotechnology Research Institute, Chinese Academy of Agricultural Sciences, Beijing 100081, People's Republic of China. Correspondence and requests for materials should be addressed to L.Q. (email: lcqian@zju.edu.cn) or B.Y. (email: binyao@caas.cn)

[*]These authors contributed equally to this work.

Supplementary Table S1. Effect of metal ions and chemical reagents (5 mM) on the activity of purified recombinant β-glucosidases from *H. insolens* Y1

Chemicals	Relative activity (%) [a]		
	HiBgl3A	HiBgl3B	HiBgl3C
None	100.0 ± 2.1	100.0 ± 2.4	100.0 ± 1.4
Mn^{2+}	127.5 ± 16	116.8 ± 2.8	123.6 ± 6.6
Mg^{2+}	110.9 ± 2. 8	77.1 ± 1.2	102.8 ± 5.4
Zn^{2+}	110.6 ± 2.8	89.9 ± 1.9	102.1 ± 1.0
Ca^{2+}	108.4 ± 3. 9	98.5 ± 1.5	102.3 ± 2.6
Cu^{2+}	106.4 ± 2. 8	83.8 ± 2.9	101.5 ± 4.6
Co^{2+}	105.6 ± 2.8	90.2 ± 6.1	103.3 ± 3.2
Cr^{3+}	104.5 ± 2.6	70.2 ± 4.5	106.9 ± 3.9
Ni^{2+}	102.1 ± 1.5	89.7 ± 2.2	102.8 ± 4.8
Pb^{2+}	100.2 ± 5.3	85.8 ± 2.9	103.9 ± 7.9
Fe^{3+}	95.3 ± 1.7	93.3 ± 2.7	93.8 ± 9.4
Ag+	28.0 ± 4.3	24.0 ± 4.9	10.6 ± 5.2
EDTA	103.1 ± 1.7	90.7 ± 2.0	101.1 ± 2.3
β-Mercaptoethanol	77.7 ± 1.1	97.4 ± 6.6	108.5 ± 5.1
SDS	45.3 ± 2.3	4.9 ± 1.2	37.8 ± 1.8

[a] Values represent the mean ± SD (n = 3) relative to the untreated control samples

Supplementary Table S2. Primers used in this study.

Primer name	Primer sequence (5′→3′) [a]
*Hi*Bgl3A-F	GGGACTAGTAACAGGCATCGCCAGGTTATCGGCAGG
*Hi*Bgl3A-R	GGGGCGGCCGCTCACGGAAGCTCAGCGCTCAACTCCAATTTC
*Hi*Bgl3B-F	GGGGAATTCCGCGTTGTCGAGCCCCGCGATC
*Hi*Bgl3B-R	GGGGCGGCCGCTCAAACCGGCTGCTTCGCCCCCTC
*Hi*Bgl3C-F	GGGACTAGTATGGGTATCTTCGGTCTCGC
*Hi*Bgl3C-R	GGGGCGGCCGCTCAAACATCAATGCTGCCCGTC
W69I-F	ACCCGAAGGCTGCAGAGATTCAGAATGCCTACGCCAA
W69I-R	TTGGCGTAGGCATTCTGAATCTCTGCAGCCTTCGGGT
F304I-F	ATGCCCGGAGATACCACGATTAACACTGGTGTCAGTTTC
F304I-R	GAAACTGACACCAGTGTTAATCGTGGTATCTCCGGGCAT
Y509T-F	GCGAACTCGGGTGAGGGGACGACAAGGGTTGACGGCAAT
Y509T-R	ATTGCCGTCAACCCTTGTCGTCCCCTCACCCGAGTTCGC
I48W-F	TCACATCGGGCGTTGGCTGGTATATGGGACCCTGTGT
I48W-R	ACACAGGGTCCCATATACCAGCCAACGCCCGATGTGA
I278F-F	ATGCCGGGCGATACAAACTTTCCCTTGTTTGGTAACAGT
I278F-R	ACTGTTACCAAACAAGGGAAAGTTTGTATCGCCCGGCAT
T484Y-F	TCCGACTCGGGAGAGAATTACCTGACAGTCGAGGGCAAC
T484Y-R	GTTGCCCTCGACTGTCAGGTAATTCTCTCCCGAGTCGGA

[a] The restriction sites are shown underlined; the mutation sites are colored gray.

Supplementary Figure S1. SDS-PAGE analysis of the recombinant β-glucosidases.

Lanes: M, the standard protein molecular weight markers; A1, B1 and C1, the purified *Hi*Bgl3A, *Hi*Bgl3B and *Hi*Bgl3C, respectively; A2, B2 and C2, the purified *Hi*Bgl3A, *Hi*Bgl3B and *Hi*Bgl3C after deglycosylation with Endo H.

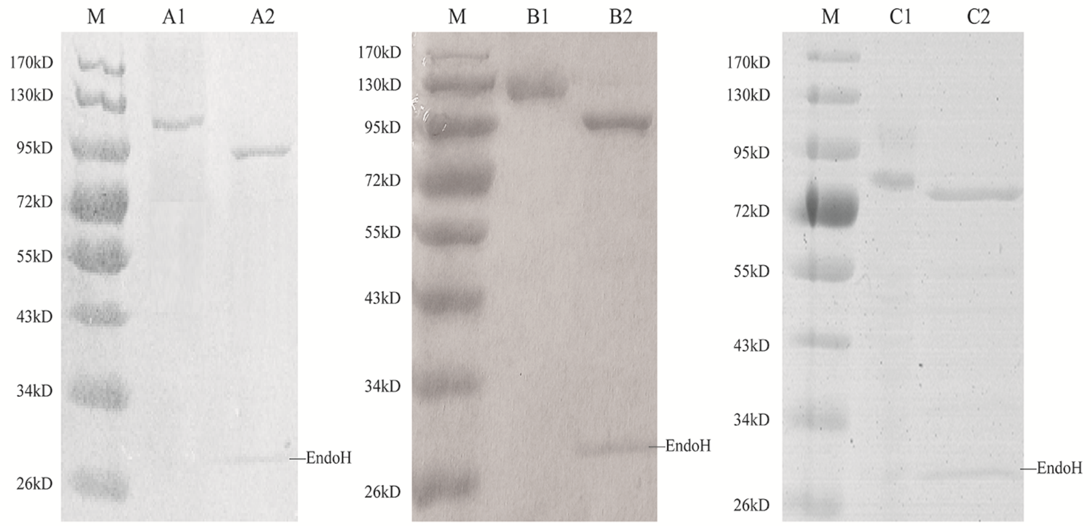

Supplementary Figure S2. The three conserved residues related to substrate recognition (shown in stick and labeled) in GH3 β-glucosidases. A, AaBgl1 from *Aspergillus aculeatus* (PDB: 4IIB); B, Cel3A from *Hypocrea jecorina*; C, *Hi*Bgl3A ; D, *Hi*Bgl3B; E, *Hi*Bgl3C.

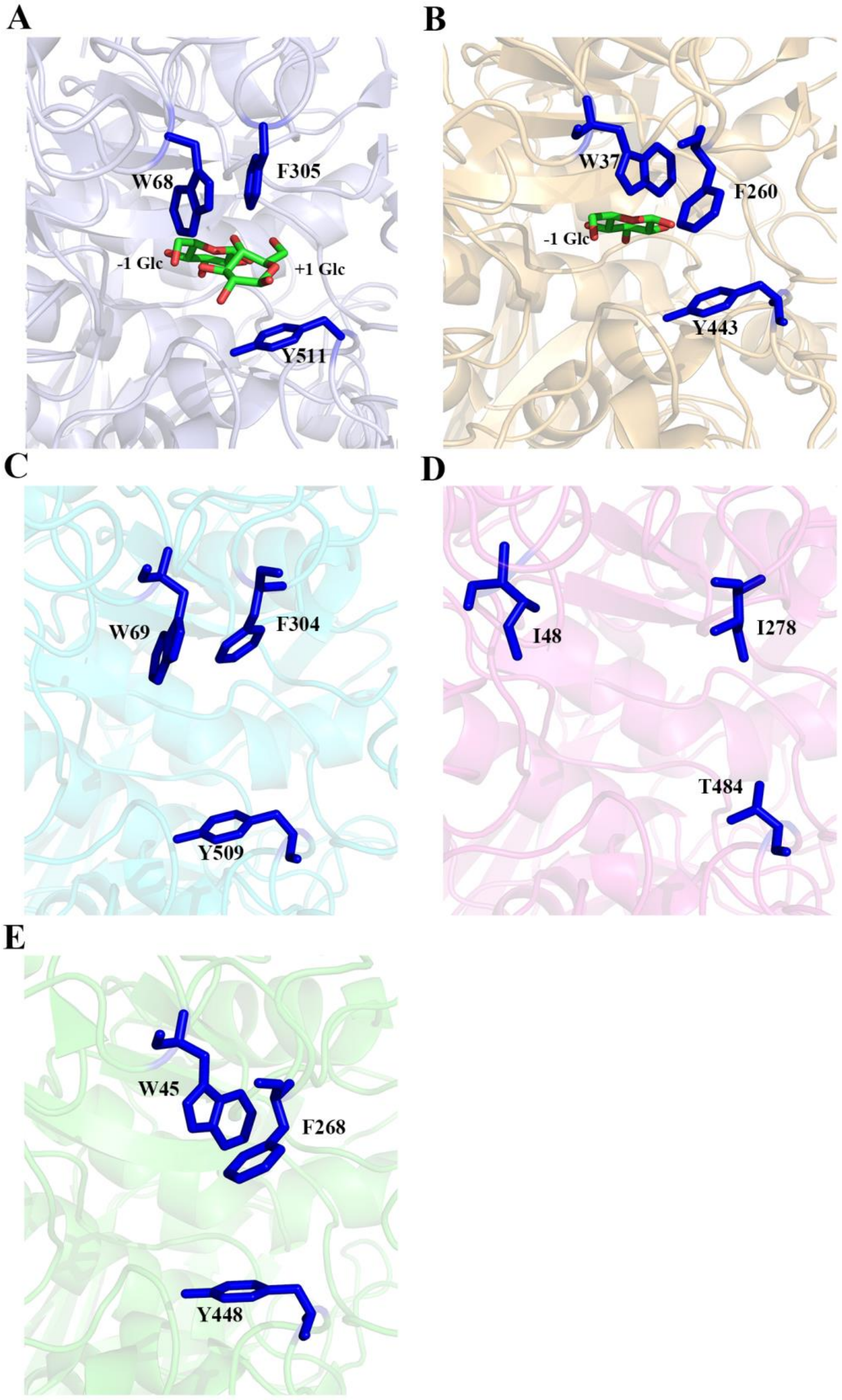

Supplement: Supplementary Information [file srep27062-s1.pdf]
